# 

**Published:** 1918-04-27

**Authors:** 


### CONTENTS.

| | PAGE |
|---|---|
| **The Future of the Medical Profession** ... ... | 67 |
| **Housekeepers, Cooks, and the Death-Rate** ... | 68 |
| **Hospital and Institutional News** ... ... ... | 69 |
| Important Developments at Leicester—A New Financial Policy—Two Fresh Enterprises—The Cost of Pensions—A Poor-Law Criticism on the Reform of Institutional Medical Relief—The Danger of Unqualified Persons on Hospital Staffs—Representation and Efficiency—The Hardships of the Ticket System—A Government Concession—A Colliery Owner's Suggestion—A Patient's Testimonial—A Blot on the'Scutcheon—Essentials to Happiness in the Home—Retirement of a Hospital Secretary—The Feeding and Care of Infants—Mr. Guy Elliston's Death—The Manchester Radium Institute—Prizes for Posters by the Wounded—The Paper Controller's Anxiety—This Week's Drug Market—To our Readers. | |
| **A Case of Huntington's Chorea** ... ... ... | 73 |
| Sir Clifford Allbutt's Clinical Lecture. | |
| **English Mission Hospital, Baghdad** ... ... | 74 |
| **The Question of Population** ... ... ... ... | 75 |
| By a Member of the A.M.S. | |
| **War Bread** ... ... ... ... ... ... ... ... | 76 |
| **The Care of Lunatics in Ireland** ... ... ... | 77 |
| Ireland's Prosperity Unexampled: Ireland for the Irish—With British Millions for Irish Cultivators! | |
| **Parliament and the Profession** ... ... ... | 78 |
| **The Editor's Letter-Box** ... ... ... ... | 79 |
| Deaths in Cottage Hospitals—The Employment of the Waiting Years. | |
| **Nursing Affairs in Ireland** ... ... ... ... | 80 |
| An Imperial Union of Nurses. | |
| **The Matrons' and Sisters' Department** ... | 81 |
| The Growth of the Nursing Profession: VIII. The Deeper and Wider Wants of the Nurse. | |
| Round the Hospitals. | |
| A Nurses' Home that Discredits Kensington—College News and Progress—Colonial Nurses' Pay and Allowances—Recruitment of Retired Nurses—Matrons-in-Chief on Dancing—A London Infirmary's Interesting Chapel. | |
| **Some Problems of To-Day** ... ... ... ... | 85 |
| The Unmarried Mother. | |
| **Matrons' and Sisters' Appointments** ... ... | 86 |
| **Annual Meetings** ... ... ... ... ... | 86 |
| **Forthcoming Events** ... ... ... ... ... | 86 |
| **The Institutional Worker** ... ... (Suppl.) | 1 |
| The Admission of Patients: Answer to March Question. | |
| Repairs and Renewals: Answer to March Question. April Question. | |
| Enquiries and Answers. | |
| Employment and Training. | |
| The Sick and in Need. | |

